# KIAA1199 promotes migration and invasion by Wnt/β-catenin pathway and MMPs mediated EMT progression and serves as a poor prognosis marker in gastric cancer

**DOI:** 10.1371/journal.pone.0175058

**Published:** 2017-04-19

**Authors:** Shuqin Jia, Tingting Qu, Xiaohong Wang, Mengmeng Feng, Yang Yang, Xuemin Feng, Ruiting Ma, Wenmei Li, Ying Hu, Yi Feng, Ke Ji, Ziyu Li, Wenguo Jiang, Jiafu Ji

**Affiliations:** 1 Laboratory of Surgery, the Affiliated Hospital of Inner Mongolia Medical University, Hohhot, China; 2 Center for Molecular Diagnosis, Key Laboratory of Carcinogenesis and Translational Research (Ministry of Education), Peking University Cancer Hospital & Institute, Beijing, China; 3 Tissue Bank, Key Laboratory of Carcinogenesis and Translational Research (Ministry of Education), Peking University Cancer Hospital & Institute, Beijing, China; 4 Department of Gastrointestinal Surgery, Key Laboratory of Carcinogenesis and Translational Research (Ministry of Education), Peking University Cancer Hospital & Institute, Beijing, China; 5 Cardiff China Medical Research Collaborative, Cardiff University School of Medicine, Cardiff, United Kingdom; University of South Alabama Mitchell Cancer Institute, UNITED STATES

## Abstract

**Background:**

KIAA1199 was upregulated in diverse cancers, but the association of KIAA1199 with gastric cancer (GC), the biological role of KIAA1199 in GC cells and the related molecular mechanisms remain to be elucidated.

**Methods:**

KIAA1199 expression was analysed by reverse transcription-polymerase chain reaction assay (RT-PCR) and immunohistochemistry (IHC) in GC patient tissue. The small hairpin RNA (shRNA) was applied for the knockdown of endogenous KIAA1199 in NCI-N87 and AGS cells. MTT, colony formation, scratch wounding migration, transwell chamber migration and invasion assays were employed respectively to investigate the role of KIAA1199 in GC cells. The potential signaling pathway of KIAA1199 induced migration and invasion was detected.

**Results:**

KIAA1199 was upregulated in GC tissue and was an essential independent marker for poor prognosis. Knockdown KIAA1199 suppressed the proliferation, migration and invasion in GC cells. KIAA1199 stimulated the Wnt/β-catenin signaling pathway and the enzymatic activity of matrix metalloproteinase (MMP) family members and thus accelerated the epithelial-to-mesenchymal transition (EMT) progression in GC cells.

**Conclusion:**

These findings demonstrated that KIAA1199 was upregulated in GC tissue and associated with worse clinical outcomes in GC, and KIAA1199 acted as an oncogene by promoting migration and invasion through the enhancement of Wnt/β-catenin signaling pathway and MMPs mediated EMT progression in GC cells.

## Introduction

Gastric cancer (GC) is one of the most lethal malignancies and the third leading cause of cancer-related mortality [1]. Thus, there is an urgent need to improve our depth of understanding on the clinical biomarkers and the molecular underpinnings that drive GC initiation, progression and metastasis. Because metastasis is a major factor responsible for poor prognosis in GC, the identification of novel molecular markers of a metastatic phenotype is a crucial challenge in GC therapy [2,3].

KIAA1199 was overexpressed in diverse cancers, such as colon cancer, breast cancer and oral squamous cell carcinoma [4–7]. The abnormal expression of molecules usually triggered a series of malignant biological initiations. In breast cancer, the expression of KIAA1199 was demonstrated to be especially upregulated in invasive breast cancer specimens and cell lines by large scale microarray and studies of breast cancer cell lines, which indicated that KIAA1199 was associated with cell proliferation, motility and apoptosis [5,8]. The role of KIAA1199 in colon cancer was extensively elucidated in recent studies. KIAA1199 had been identified not only to be associated with clinicopathological parameters and 5 year-overall survival rate in colon cancer but also as an essential inducer for cell proliferation, migration and invasion by complex signaling pathways[9–11]. Proteomics analysis by two-dimensional gel electrophoresis and mass spectrometry assay revealed KIAA1199 was one novel protein that expressed abnormally in oral cancer, which was then demonstrated by Realtime-PCR assay [6]. Furthermore, the latest report also indicated KIAA1199 as a newly identified protein which probably played significant role in pancreatic ductal adenocarcinoma [7]. Besides, over expression of KIAA1199 was also detected in GC by Chivou *et al* and Matsuzaki *et al* [12,13]. Whereas, further research on KIAA1199 in GC was rarely reported and how the KIAA1199 affected GC progression was still unknown.

In this study, the expression of KIAA1199 in GC patient tissues verified thatKIAA1199 was associated with the depth of invasion, distant metastasis and the overall survival rate in GC patients. Furthermore, on the basis of clinicopathological parameters analysis, two selected GC cell lines were utilized to validate that KIAA1199 was involved in the malignant biological progression including cell proliferation, especially more dominant in cell migration and invasion. Finally, we elucidated that the molecular mechanism of KIAA1199 on GC cell migration and invasion was related to the EMT progression which induced by Wnt/β-catenin signaling pathway and the activation of MMPs.

## Materials and methods

### Patients and gastric tissue specimens

A total of 321 paraffin-embedded and 123 paraffin-sectioned gastric cancer tissues were collected from GC patients from Peking University Cancer Hospital between 1998 and 2008. Furthermore, 15 surgically removed frozen GC samples in 2014 were obtained from the BioBank of Peking University Cancer Hospital. Some patients received chemotherapy or radiation therapy before surgery. All human samples were obtained through written informed consent from patients and the ethics committee of Peking University Cancer Hospital approved these tissues for research use. The following clinicopathological information was obtained from patient data. GC staging was classified according to 1997 Union for international cancer control (UICC)-TNM criteria.

### Immunohistochemistry

For immunohistochemistry, 4 μm-thick sections cut from the FFPE tissue blocks were deparaffinized and rehydrated using xylene and a graded ethanol washes. Antigen retrieval was performed in 10mmol sodium citrate buffer (pH 6.0) for 20 minutes, and endogenous peroxidase activity was blocked with 3% hydrogen peroxide for 15 minutes. The sections were then blocked with normal sheep serum at room temperature for 90minutes, and incubated with KIAA1199 Abcam antibody (Abcam, ab-76849) diluted at 1:100 overnight at 4°C. The sections were incubated at room temperature for 1 hour with the secondary antibody and then were incubated with peroxidase substrate solution. Finally, the sections were counterstained with hematoxylin followed by being dehydrated in ethanol and cleared with xylene. GC specimens were defined as KIAA1199-negative expression when less than 20% cancer cells were stained or KIAA1199-positive expression when 20% or more cancer cells were stained. Negative control was prepared by substituting PBS for the primary antibody.

### Cell lines and cell culture

All the GC cell lines were provided by the Peking University Cancer Hospital & Institute. SGC-7901, BGC-803, NCI-N87 and AGS cell lines were cultured in Dulbecco’s modified Eagle medium (DMEM; MAC GENE) supplemented with 10% fetal bovine serum (FBS; HyClone) and BGC-823 cell line was cultured in DMEM supplemented with 5% FBS. All the cell lines were maintained at 37°C, 5%CO_2_.

### RNA interference

The small hairpin RNA (shRNA) was used for the knockdown of endogenous KIAA1199 in NCI-N87 and AGS cells. The target sequence was from the previous published paper [14]. The target sequence was: 5’-CGAATGAAGATCATCAAGAAT-3’, and GV248 was used as sh-control. Cells with depleted endogenous KIAA1199 expression were selected by being cultured in puromycin at the final selection concentration of 2μg/ml.

### RNA extraction and RT-PCR

Total RNA was extracted from tissue samples and cell lines using the Trizol reagent (Ambion,15596–026) according to the manufacturer’s instructions. Reverse transcription-polymerase chain reaction was used to determine the mRNA level of KIAA1199 in the gastric carcinoma tissue and its adjacent noncancerous tissue. GAPDH was used as a control. PCR was performed in PCR reaction mix with initial heating at 94°C for 5 minutes, followed by 30 cycles of 94°C for 30 seconds, 60°C for 30 seconds and 72°C for 30seconds, then with a final extension step of 72°C for 10 minutes. The primers for KIAA1199 were KIAA1199, forward: TGCCACGGTCTATTCCATC, reverse: TCCTTTACCAACCCCAATG; The primers for GAPDH were GAPDH, forward: GCATCCTGGGCTACACT, reverse: CACCACCCTGTTGCTGT.

### Quantitative real-time PCR

Quantitative real-time PCR was performed with the ABI PRISM 7500 Sequence Detection System using the SYBR Green method. The mRNA levels of all detected genes were normalized to GAPDH. Specific primers used in PCR amplification were as follows: KIAA1199, forward: TGCCACGGTCTATTCCATC, reverse: TCCTTTACCAACCCCAATG; β-catenin, forward: ACGGAGGAAGGTCTGAGGAG, reverse: AGCCGCTTTTCTGTCTGGTT; c-Myc, forward: TGGTCTTCCCCTACCCTCTCAAC, reverse: GATCCAGACTCTGACCTTTTGCC;cyclinD1,forward:GATGCCAACCTCCTCAACGA,reverse:GGAAGCGGTCCAGGTAGTTC; MMP2, forward: AGTTTCCATTCCGCTTCCAG, reverse: CGGTCGTAGTCCTCAGTGGT; MMP7, forward: CATGATTGGCTTTGCGCGAG, reverse: AGACTGCTACCATCCGTCCA; MMP9, forward: CCAACTACGACACCGACGAC, reverse: TGGAAGATGAATGGAAACTGG; MMP14, forward: AGCCATATTGCTGTAGCCAG, reverse: GTTGTCTCCTGCTCCCCCT; Slug, forward: CTACAGCGAACTGGACACACA, reverse: GCCCCAAAGATGAGGAGTATC; Snail, forward: TCCAGAGTTTACCTTCCAGCA, reverse: CTTTCCCACTGTCCTCATCTG; Twist, forward: GTCCGCAGTCTTACGAGGAG, reverse: GTCTGAATCTTGCTCAGCTTGTC; Vimentin, forward: GGACCAGCTAACCAACGACA, reverse: AAGGTCAAGACGTGCCAGAG; GAPDH, forward: GCATCCTGGGCTACACT, reverse: CACCACCCTGTTGCTGT.

### Western blotting

Cells were lysed completely in lysis buffer (50mM Hepes pH7.5, 150mM NaCl, 2mM EDTA, 2mM EGTA, 1% TritonX-100, 50mM NaF, 5mM Sodiun Pyrophosphate, 50mM Sodium β-glycerophosphate, 1mM DTT, 1mM PMSF, 10μg/ml Leupeptin, 10μg/ml Aprotinin) at 4°C. The protein content was determined using a BCA Protein Assay Kit (Thermo, USA). Total proteins were separated by SDS-PAGE and then transferred onto PVDF membranes (Millipore, USA). The blotted membranes were incubated with primary antibodies and then with corresponding secondary antibody of the primary antibody. Antibody against GAPDH (Santa Cruz, sc-166545) was purchased from Santa Cruz. The KIAA1199 (Proteintech, 21129-1-AP) antibody and Lamin B1 (Proteintech, 66095-1-Ig) antibody were purchased from Proteintech. The β-cateninantibody was provided by Division of Gastrointestinal Cancer Translational Research Laboratory (Peking University Cancer Hospital & Institute).

### Cytoplasm and nuclear extraction

1×10^7^ cells were prepared and washed with PBS for twice. The cell pellets were resuspended gently in 300 μl CER I (10mM Hepes pH7.9, 1.5mM MgCl_2_, 10%glycerol, 10mM KCl, 0.34M sucrose, 0.5mM DTT, 1mM PMSF, 10μg/ml Leupeptin, 10μg/ml Aprotinin) before being vortexed vigorously for 20 seconds and was then left on ice for 10 minutes. After the addition of NP-40 at the final concentration of0.3%, the cell resuspension was vortexed again vigorously for 10 seconds and was left on ice for 1 minute, and such step was repeated for thrice followed by centrifugation at 12000 rpm for 10 minutes. The supernatants were then collected as the cytoplasmic extraction. The pellets were suspended with 60 μl lysis buffer (50mM Hepes pH7.5, 150mM NaCl, 2mM EDTA, 2mM EGTA, 1% TritonX-100, 50mM NaF, 5mM Sodiun Pyrophosphate, 50mM Sodium β-glycerophosphate, 1mM DTT, 1mM PMSF, 10μg/ml Leupeptin, 10μg/ml Aprotinin) before being vortexed for 20 seconds and was then left on ice for 10 minutes, and this step was repeated for quartic. Finally, the lysate was centrifuged at 12000 rpm for 10 minutes and supernatants were collected as the nuclear extraction.

### Cell proliferation assay

The MTT assay was performed to assess cell proliferative activity. NCI-N87 and AGS cells were seeded into 96-well plates at 1×10^3^ per well and incubated at 37°C with 5% CO_2_ for 24 hours, 48 hours, 72 hours and 96 hours. At the detection time points, MTT solution was added to each well and the plates were incubated for another 4 hours. The formazan crystals were then dissolved with 100 μl dimethyl sulfoxid per well. The absorbance of at least 3 individual wells of one cell type at each time point was read using a microplate reader (Bio-Rad, USA).

### Colony formation

For colony formation assay, 500 of NCI-N87 and AGS cells were seeded in triplicate in 60-mm dishes. After appropriate time of growth, the cells were washed with PBS for three times followed by being fixed with 4% paraformaldehyde for 20 minutes. The cells were then stained with 0.1% crystal violet for 20 minutes, and were finally washed with PBS for three times. Photographs were captured and the numbers of the cell clones in 3 independent samples were counted.

### Scratch wounding migration assay

Scratch wounding migration assay was detected by the IncuCyte HD system (IncuCyte ZOOM, Essen BioScience, USA). NCI-N87 and AGS cells were grown in 96-well culture plates until the formation of a monolayer. Cell layers were scraped with a pin block and then incubated at 37°C. Photographs were captured at set time points by IncuCyte HD system. The migration distance was measured at each time point in 3 independent samples.

### Transwell chamber migration and invasion assay

Transwell chamber migration assay was measured using a transwell chamber with 8μm filter inserts (BD Biosciences, USA) without Matrigel. For transwell chamber-based invasion assay, transwell inserts with 8 μm filter were pre-coated with 50 μg of Matrigel (Becton Dickinson, San Jose, CA).5×10^4^ cells were added to the inserts containing serum-free DMEM medium. The lower chamber was filled with 600 μl DMEM medium with 10% FBS. After 24 hours’ incubation for AGS cells (migration and invasion) and 48 hours’ (migration) or 72 hours’ (invasion) incubation of NCI-N87 cells, the inside of the inserts was cleaned thoroughly with a cotton swab to remove any non-migrated or non-invasive cells, and the cells which had migrated through the porous membrane and invaded into the Matrigel were fixed with 4% paraformaldehyde for 20 minutes, and were then stained with 0.1% crystal violet for 20 minutes. Photographs were captured and the migrated cells were counted in at least 3 random fields.

### Statistical analysis

The association of KIAA1199 mRNA and protein expression with clinicopathological features in GC patients was analysed by Chi-square test and Kruskal-Wallis test. Cox regression analysis was utilized to estimate the relative risks of death associated with KIAA1199 protein expression. The overall survival curve was analysed by the Kaplan-Meier method. Other data were analysed using Student’s *t*-test. Statistical analysis was carried out using the SPSS 21.0 software (SPSS Inc., Chicago, IL, USA). All data were represented as mean value ± S.D. A two-tailed P < 0.05 was considered statistically significant.

## Results

### KIAA1199 is frequently overexpressed in primary GC

**Fig 1:**
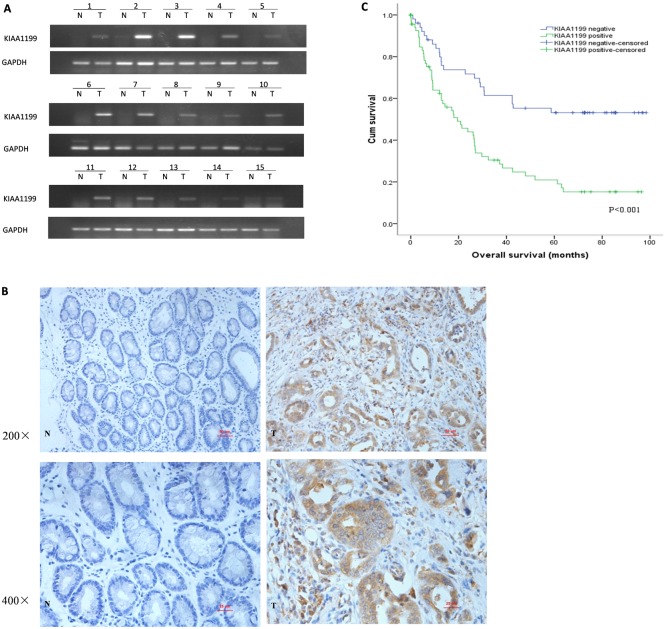
KIAA1199 expression in primary GC tissues and survival curve for KIAA1199 expression in patients with GC. (**A**)The mRNA expression of KIAA1199 in adjacent noncancerous tissue and GC samples by RT-PCR. (**B**) KIAA1199 expression by immunohistochemical staining. N: adjacent noncancerous tissue; T: gastric cancer tissue. Original magnification: 200× and 400×. (**C**) Kaplan-Meier survival curves analysis of overall survival for all patients with KIAA1199 negative and positive GC tissue.

RT-PCR was applied for semi-quantitative analysisonKIAA1199 mRNA expression in primary GC. Initial screening of surgically resected CGs from 15 patients revealed that KIAA1199 was upregulated in 13 cases (Fig 1A). To further confirm this result, we also examined the protein expression of KIAA1199 in primary GC by immunohistochemistry (IHC) with an anti-KIAA1199 antibody. As shown in Fig 1B, KIAA1199 was seldom expressed in adjacent noncancerous tissue, but was highly expressed in the cytoplasm of GC tissue cells.

### Association of KIAA1199 expression level with clinicopathological parameters in GC patients

To investigate the clinical role of KIAA1199 in GC, we assessed the correlations between KIAA1199 expression and clinicopathological parameters, including sex, depth of invasion (T-staging), lymph node status, distant metastasis (M-staging), TNM staging, tumor differentiation and clinical outcome of GC patients. We first utilized Realtime-PCR to detect the KIAA1199 mRNA expression in 321 GC patient tissues. As shown in Table 1, statistical analysis showed that KIAA1199 mRNA expression was correlated with the clinical characteristics regarding depth of invasion (T-staging), distant metastasis (M-staging) and TNM staging (T-staging: P<0.001; M-staging: P<0.05; TNM: P<0.001) of GC patients. The protein expression of KIAA1199 was also detected by IHC. The IHC analysis on the biopsies from123 GC patients showed that KIAA1199 protein expression had no significant effect on such clinical parameters regarding age, sex, vascular invasion and differentiation, but was correlated with depth of invasion, lymph node status (N-staging), metastasis and TNM staging (T-staging: P<0.01; N-staging: P<0.01; M-staging: P<0.05; TNM: P<0.01), which was mostly consistent with the KIAA1199 mRNA expression clinical characteristics analysis in GC patients (Table 2). As shown in Table 3, the multivariate analysis of overall survival (OS) indicated that KIAA1199 expression was an essential independent marker for poor prognosis (P<0.01). Kaplan-Meier survival curves showed that the OS of the patients with KIAA1199 expression was worse compared to the patients with no expression of KIAA1199 in GC (P<0.001, Fig 1C).

**Table 1:** Relationship between KIAA1199 mRNA expression and clinicopathological features in patients with GC.

Clinicopathological Features	Cases	KIAA1199 expression	*P*
		Median (Q1, Q3)	
Gender			
Male	229	58.86(4.36–90.64)	0.616
Female	92	50.00(3.50–59.60)	
Depth of invasion			
T1-2	42	37(3.19–30.60)	0.000
T3-4	271	19(4.39–96.85)	
Lymph node status			
N0	69	22.2(4.5–100.1)	0.46^a^
N1-3	246	20.05(4.28–86.95	
Distant metastasis			
M0	272	20(4.44–82.63)	0.028
M1	40	35.6(3.50–104.90)	
TNM staging			
TNM1-2	84	19.2(4.4–65.1)	0.000
TNM3-4	228	20.76(4.35–91.39)	
Differentiation			
High-medium	6	31.9(14.6–72.8)	
Medium	62	10.6(3.8–87.6)	0.09^b^
Medium-low	82	39.4(7.6–142.8)	0.002^b^
Low	136	14.3(3.2–51.1)	0.199^b^

^a^ Compared with “N0”.

^b^ Compared with “High-medium”.

**Table 2:** Relationship between KIAA1199 protein expression and clinicopathological features in patients with GC.

Clinicopathological Features	Cases	KIAA1199 expression		χ^2^	*P* ^a^
		Negative (%)	Positive (%)		
Gender					
Male	78	30(38.5%)	48(61.5%)	0.792	0.374
Female	45	21(46.7%)	24(53.3%)		
Age (years)					
≤60	57	28(49.1%)	29(50.9%)	2.568	0.109
>60	66	23(34.8%)	43(65.2%)		
Vascular invasion					
Absent	44	23(52.3%)	21(47.7%)	3.546	0.06
Present	75	26(34.7%)	49(65.3%)		
Depth of invasion					
T1-2	34	22(64.7%)	12(35.3%)	10.458	0.001
T3-4	89	29(32.6%)	60(67.4%)		
Lymph node status					
N0	29	19(65.5%)	10(34.5%)	9.046	0.003
N1-3	94	32(34.0%)	62(66.0%)		
Distant metastasis					
M0	109	49(45.0%)	60(55.0%)	4.808	0.028
M1	14	2(14.3%)	12(85.7%)		
TNM staging					
I	9	5(55.6%)	4(44.4%)	16.463	0.001^b^
II	25	18(72.0%)	7(28.0%)		
III	78	27(34.6%)	51(65.4%)		
IV	11	1(9.1%))	10(90.9%)		
Differentiation					
High	0	0(0.0%)	3(100.0%)	6.058	0.195
High-medium	5	1(20.0%)	4(80.0%)		
Medium	17	8(47.1%)	9(52.9%)		
Medium-low	27	8(29.6%)	19(70.4%)		
Low	71	34(47.9%)	37(52.1%)		

^a^ Chi-square test,

^b^ Kruskal-Wallis test.

**Table 3:** Univariate and multivariate Cox regression analysis for overall survival of patients with GC.

Clinicopathological Features	Univariate	*P*	Multivariate	*P*
	Overall survival rate (mean ± S.E)		RR 95% CI	
KIAA1199 expression		0.000		0.002
Negative	61.795±5.763		0.437(0.261,0.731)	
Positive	31.390±4.123			
Vascular invasion		0.000		0.017
Absent	65.040 ±5.957		0.506(0.290, 0.885)	
Present	31.823 ±4.170			
Depth of invasion		0.003		0.045
T1	89.218 ±7.407		0.132(0.018, 0.959)	
T2-4	41.212 ±3.784			
Lymph node status		0.001		0.776
N0	67.527 ±6.998		0.907(0.0.460,1.789)	
N1-3	37.029 ±4.032			
Distant metastasis		0.000		0.001
M0	49.548 ±3.981		0.297(0.149,0.589)	
M1	9.101 ±2.504			
Differentiation		0.221		
High	27.120±9.993		-	-
High-medium	59.704±14.104			
Medium	52.419±8.831			
Medium-low	52.914±8.516			
Low	38.185±4.562			

### Knockdown KIAA1199 reduced cellular proliferation in GC cells

**Fig 2:**
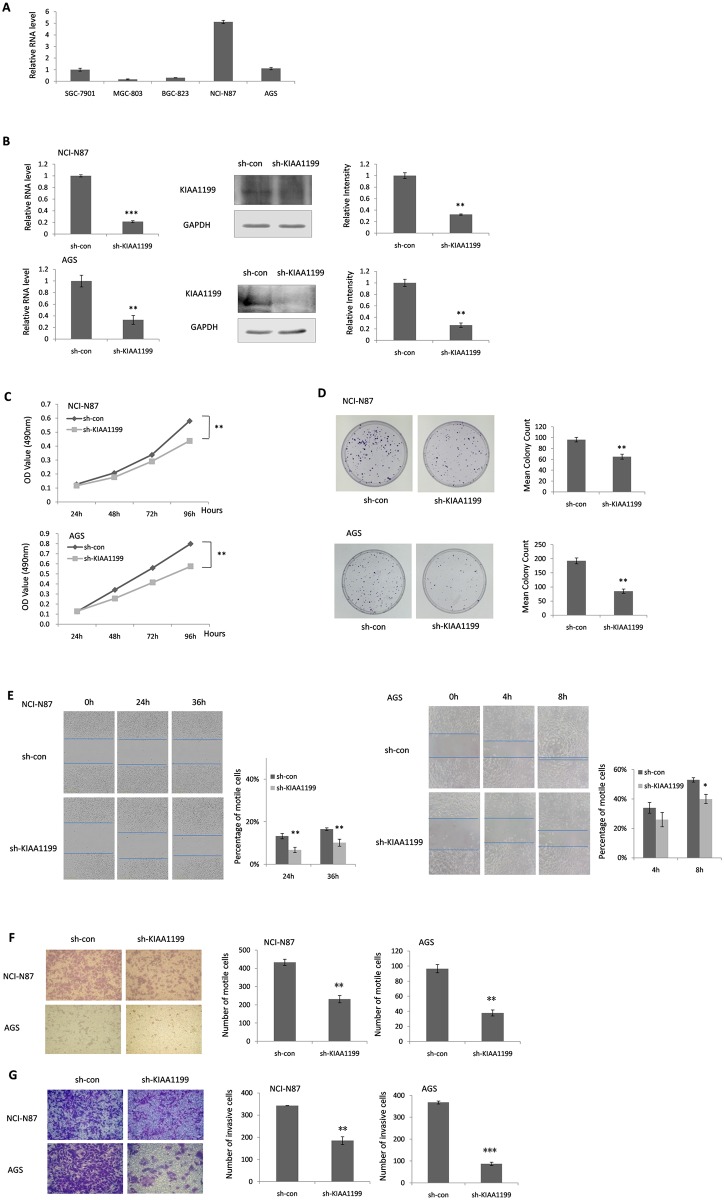
The function of KIAA1199 in NCI-N87 and AGS GC cells. (**A**) The mRNA expression of KIAA1199 in different GC cell lines by Realtime-PCR. (**B**) The mRNA and protein expression of KIAA1199 were significantly reduced in NCI-N87 and AGS GC cells transfected with sh-KIAA1199, detected by Realtime-PCR and Western blotting. Sh-control served as the negative control. (**C**) The MTT proliferation assay and (**D**) colony formation assay revealed a growth inhibitory effect of knockdown of KIAA1199 in NCI-N87 and AGS GC cells. (**E**) Knockdown of KIAA1199 reduced wound closure in scratch wounding migration assay in NCI-N87 and AGS GC cells. Photograghs were taken at 24 and 36 h in NCI-N87 cells, 4 and 8 h in AGS cells. (**F**) Knockdown of KIAA1199 decreased migration though Transwell chamber migration assay. (**G**) KIAA1199 knockdown inhibited the GC cell invasion ability though Transwell chamber invasion assay. The data were shown as mean ± SD. *P<0.05, **P<0.01,***P<0.001.

In order to explore the role of KIAA1199 in gastric cancer cell proliferation, we assessed the KIAA1199 expression level in five gastric cancer cell lines (Fig 2A) and utilized a loss of function approach in two KIAA1199 high expression GC cell types—NCI-N87 and AGS. The KIAA1199 expression of two selected GC cell lines which infected with a lentivirus expressing anti-KIAA1199 small hairpin RNA (shRNA) were confirmed by Realtime-PCR and Western blotting (Fig 2B). The effect of KIAA1199 on the proliferation of two GC cell lines was examined through MTT assay and clone formation assay. As shown in Fig 2C and 2D, both of these two assays revealed that KIAA1199 knockdown suppressed the proliferation of NCI-N87 and AGS cells compared to their respective sh-control cells (P<0.01).

### KIAA1199 promoted both GC cell migration and invasion

Migration and invasion are proved as two essential factors which accelerate the malignant biological occurrence. The occurrence of these two malignant biological processes is usually accompanied by some clinicopathological parameters changes. Since KIAA1199 expression was significantly associated with depth of invasion (T3+T4 staging) and distant metastasis (M1 staging) in patients with GC, it was likely that KIAA1199 played important roles in GC cell migration and invasion. As expected, scratch wounding and transwell chamber migration assays exhibited that the downregulation of KIAA1199 inhibited GC cells migration (NCI-N87, scratch wounding assay: P<0.01, transwell chamber migration assay: P<0.01; AGS, scratch wounding assay: P<0.05, transwell chamber migration assay: P<0.01)(Fig 2E and 2F). Furthermore, the effect of KIAA1199 on GC cell invasion was also investigated. As shown in Fig 2G, the invasive ability was impaired in sh-KIAA1199 GC cells compared to that of in sh-control cells (NCI-N87, P<0.01; AGS, P<0.001) in transwell chamber invasion assay.

### KIAA1199induced EMT progression by upregulatingWnt/β-catenin signaling and MMPs in GC cells

**Fig 3:**
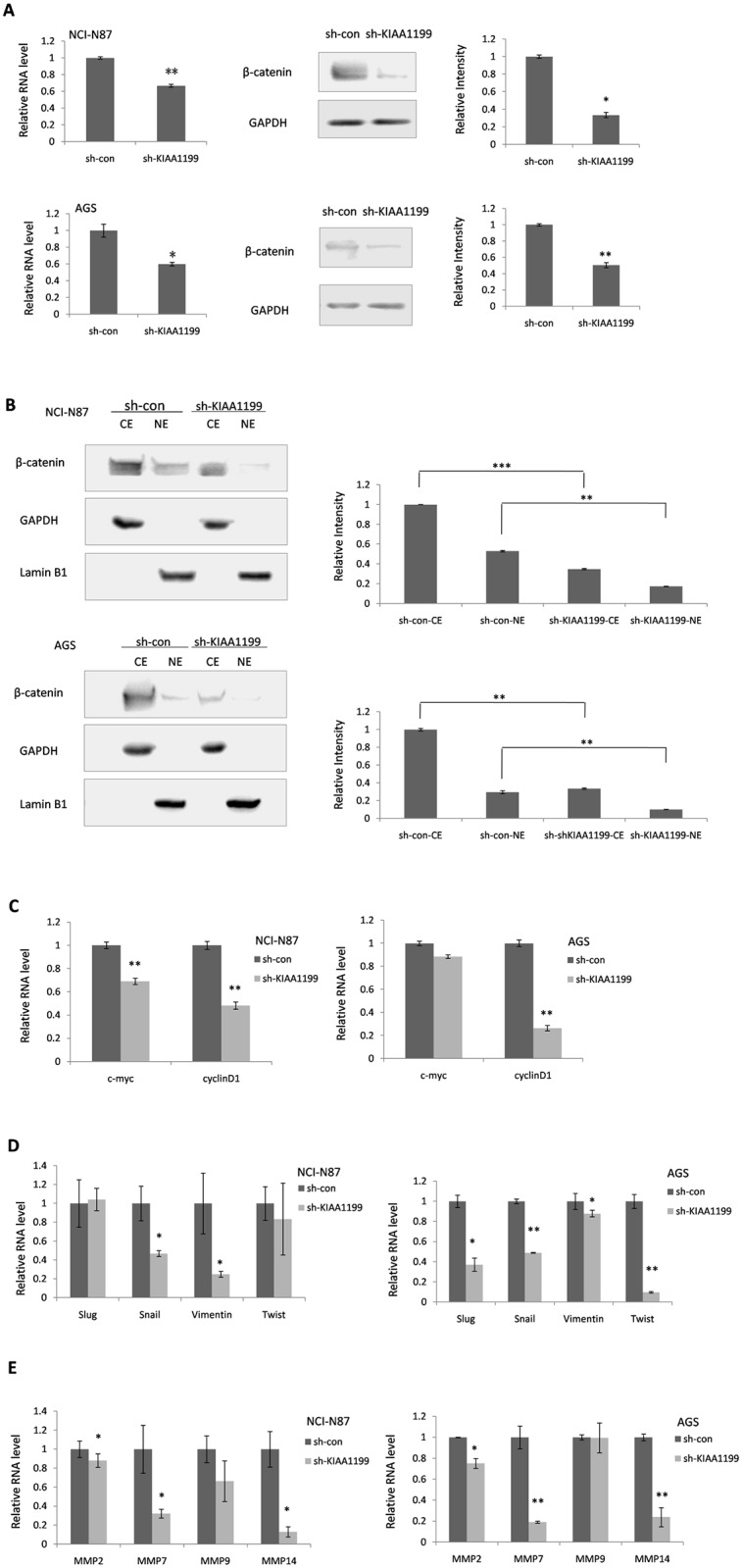
KIAA1199 induced EMT progression by upregulating Wnt/β-catenin signaling and MMPs in GC cells. (**A**) The mRNA and protein expression of β-catenin were significantly reduced in KIAA1199 knockdown NCI-N87 and AGS GC cells compared with sh-control cells respectively, detected by Realtime-PCR and Western blotting. (**B**) The nucleus expression of β-catenin was also decreased in KIAA1199 knockdown NCI-N87 and AGS GC cells, detected by Western blotting. (**C**)The expression of c-myc and cyclinD1 was detected in KIAA1199 knockdown NCI-N87 and AGS GC cells by Realtime-PCR, and cyclinD1 expression was significantly reduced. (**D**) The expression of the mesenchymal marker Vimentin, EMT inducer Slug, Snail and Twist in KIAA1199 knockdown and sh-control NCI-N87 and AGS GC cells were analysed by Real-time PCR. (**E**) The expression of matrix metalloproteinase family member MMP2, MMP7, MMP9 and MMP14 were examined by Realtime-PCR in NCI-N87 and AGS GC cells. The data were shown as mean ± SD. *P<0.05, **P<0.01,***P<0.001.

To further investigate the mechanism of KIAA1199 induced GC cell invasion and metastasis, we examined some pathways that are involved in cell invasion and metastasis. As shown in Fig 3A, knockdown of KIAA1199 reduced β-catenin expression in NCI-N87 and AGS cells. The decreased expression of β-catenin abolished itself accumulation in cytoplasm which caused less β-catenin to enter the nucleus (Fig 3B), thus inhibited the initiation of Wnt/β-catenin signaling pathway. Therefore, we also detected the expression of two main downstream target genes involved in Wnt/β-catenin signaling pathway. As shown in Fig 3C, the expression of c-myc and cyclinD1 were both decreased, especially cyclinD1. According to this result, we proposed that KIAA1199 was possibly associated with invasion and metastasis through Wnt/β-catenin signaling pathway.

The EMT is one of the major complex processes mediated by Wnt/β-catenin signaling pathway which plays important role in cancer invasion and metastasis [15,16]. The inducers which have been demonstrated to be involved in EMT initiation during cancer progression and metastasis can be classified as cellular factors, transcriptional factors and matrix metalloproteinases. The EMT-related molecules were detected in Fig 3D, and the mRNA expression of Snail and Vimentin were decreased by KIAA1199 knockdown in NCI-N87 cells (P<0.05). Additionally, the mRNA expression levels of four typical EMT-related molecules were all decreased significantly (Slug, P<0.05; Snail, P<0.01; Vimentin, P<0.05; Twist, P<0.01) in AGS cells. Furthermore, MMP family, which has previously been shown to induce EMT in variety of cancer, was also detected in Fig 3E. The results revealed that except for MMP9, the expression of MMP2, MMP7 and MMP14 were suppressed in KIAA1199 knockdown NCI-N87 and AGS cells(NCI-N87: MMP2, MMP7,MMP14, P<0.05; AGS: MMP2, P<0.05; MMP7,MMP14, P<0.01).

## Discussion

In order to identify new biomarkers for the improvement of diagnosis strategies and targeted therapies, better understand biology and the molecular profiles of GC is an essential work. In this study, we first examined the mRNA and protein expression levels of KIAA1199 by RT-PCR and immunohistochemistry in 15 surgically resected GC cases and 123 paraffin section samples (1997–2008) respectively. The results indicated that KIAA1199 was strongly expressed in GC tumors in comparison to normal gastric tissue. Based on these data, we also verified the KIAA1199 mRNA and protein expression in 321 cases (2004–2007) and 123 cases of GC patients by Realtime-PCR and immunohistochemistry respectively to further investigate the association of KIAA1199 expression with clinicopathological parameters changes. Statistical analysis showed that KIAA1199 expression was not significantly correlated with such clinical parameters regarding age, sex, vascular invasion and pathological differentiation degrees (P>0.05). Interestingly, significant correlations were observed between KIAA1199 expression and depth of invasion, lymph node status, distant metastasis and TNM staging of GC patients. The statistical analysis on the relationship between these two main meaningful expression levels of KIAA1199 (mRNA and protein) and clinicopathological parameters were mostly consistent with each other. Another study on GC by Matsuzaki *et al* was also implicated the overexpression of KIAA1199 in cancer tissue and the relationship between KIAA1199 and lymph node metastasis, which was in agreement to our result [13]. Previous studies in other kinds of cancer also revealed the abnormal expression of KIAA1199 in cancer tissue, such as breast cancer, colon cancer and pancreatic ductal adenocarcinoma [5,7,10]. Additionally, similar to our result, the expression of KIAA1199 was also significantly associated with tumor invasion depth, lymph node metastasis and TNM staging, which was demonstrated in clone cancer study. Furthermore, KIAA1199 was indicated as a prognostic factor and novel therapeutic target for clone cancer because it was also related to the survival time of patients. These previous results in clone cancer were partly in consistent with the pathological parameter analysis and the overall survival rate of GC in this study [4,9,11].

According to the significant changes of clinicopathological parameters, we further explore the role of KIAA1199 in GC progression. KIAA1199 knockdown reduced cell proliferation, migration and invasion in NCI-N87 and AGS GC cells. Other related studies revealed that KIAA1199 could promote cell proliferation, mobility and invasion in breast cancer and colon cancer by different researchers in recent years [5,11,14]. However, the mechanism of the specific molecule inducing these biological processes in different cancer is complicated and it still needs to be further investigated. Several previous reports found KIAA1199 was involved in EGFR and Wnt signaling pathways in breast cancer, cervical cancer and colon cancer [14,17], nevertheless, the mechanism of KIAA1199 in GC still remains unknown.

In the present study, we detected several typical signaling pathways in GC cells especially the ones which participated in cell migration and invasion based on the clinicopathological characteristics. As we all known, β-catenin was an important component in Wnt signaling pathway. Once β-cateninin the cytoplasm accumulating to a certain content, it then translocated from the cytoplasm to the nucleus where it regulated target genes which were involved in cell migration, invasion and metastasis. Therefore, the expression level of cytoplasm β-catenin was of importance for its biological functions. The abnormal expression of β-catenin had been reported to be associated with the development and progression of several types of cancers, such as colon cancer, breast cancer, gastric cancer and other malignant tumors [18–20]. Clinicopathological parameters also demonstrated that β-catenin was a risk factor for TNM staging, distant metastasis and lymph node metastasis [21]. According to our results, knockdown of KIAA1199 not only resulted in decreased expression of β-catenin, but inhibited the translocation of β-catenin from cytoplasm to nucleus in GC cells. The decreased expression of two main downstream targets of Wnt/β-catenin pathway was also detected in KIAA1199 knockdown GC cells in comparison to sh-control cells. These results suggested that KIAA1199 could potentially promote GC cell migration and invasion by the indirect up-regulation of Wnt/β-catenin pathway. This finding was in agreement to the result that had been reported in colon cancer, which explained the association of KIAA1199 with cell proliferation [14]. Moreover, EMT is another key factor to promote cancer invasion and metastasis. In recent studies, one of the unveiled regulatory patterns of various signaling pathways, including Erk, JNK, Smad, is through the promotion of cell migration and invasion by triggering EMT progression [22–24]. Wnt/β-catenin pathway was also identified as one of these essential signaling pathways. Our results demonstrated that knockdown of KIAA1199 in two GC cells significantly reduced the expression of four typical EMT-related molecules. It implicated that one pathway of KIAA1199 induced migration and invasion was related to the EMT progression which mediated by Wnt/β-catenin pathway. EMT is a complicated biological process and can be upregulated by various inducers except these signaling pathways mentioned above. In our study, we found that three MMP family members (MMP2, MMP7 and MMP14) which had been verified to induce EMT progression in different types of cancer were decreased in two KIAA1199 knockdown GC cells. This result indicated that another potential mechanism of KIAA1199 inducing migration and invasion was due to the increased MMPs expression, which could also promote EMT progression.

## Conclusions

In summary, we have identified that KIAA1199 was upregulated in GC tissues and its expression was significantly correlated with depth of invasion, lymph node metastasis, distant metastasis and TNM staging. In addition, high expression of KIAA1199 correlated with worse overall survival of GC patients. More importantly, this is the first evidence that comprehensively confirmed the biological effects of KIAA1199 in GC progression, including the acceleration of cell proliferation, migration and invasion. The downstream mechanism of KIAA1199 altering GC cell behaviors is potentially via the activation of EMT progression triggered by Wnt/β-catenin signaling pathway and MMPs. This is the first study to investigate the impact of KIAA1199 on important cellular traits of GC cells, and such effect was supported by the clinical evidence. Several key molecules which closely related to EMT progression and a profound regulatory mechanism of KIAA1199 in GC progression were highlighted as potential biomarkers or therapeutic targets for GC therapy.

## Supporting information

S1 File Supporting data excel file. All data that make up the tables and figures within this paper is stored in this file as pre PLOS One requirements.
(XLS)

## Abbreviations

EMT: Epithelial-to-mesenchymal transition
GC: Gastric cancer
MMP: Matrix metalloproteinase
RT-PCR: Reverse transcription-polymerase chain reaction assay
shRNA: Small hairpin RNA
TNM: Tumor node metastasis
